# Mixed forests with native species mitigate impacts of introduced Douglas fir on soil decomposers (Collembola)

**DOI:** 10.1002/eap.70034

**Published:** 2025-05-04

**Authors:** Jing‐Zhong Lu, Junbo Yang, Christian Bluhm, Estela Foltran, Carmen Alicia Rivera Pérez, Jonas Glatthorn, Christian Ammer, Norbert Lamersdorf, Andrea Polle, Matty Berg, Anton M. Potapov, Stefan Scheu

**Affiliations:** ^1^ J.F. Blumenbach Institute of Zoology and Anthropology University of Göttingen Göttingen Germany; ^2^ Senckenberg Museum for Natural History Görlitz Görlitz Germany; ^3^ Guangxi Key Laboratory of Plant Conservation and Restoration Ecology in Karst Terrain Guangxi Institute of Botany, Guangxi Zhuang Autonomous Region and Chinese Academy of Sciences Guilin China; ^4^ Department of Soils and Environment Forest Research Institute Baden‐Württemberg Freiburg Germany; ^5^ Soil Science of Temperate Ecosystems University of Göttingen Göttingen Germany; ^6^ Forest Botany and Tree Physiology University of Göttingen Göttingen Germany; ^7^ Institute of Biodiversity and Environmental Sciences Carl von Ossietzky University of Oldenburg Oldenburg Germany; ^8^ Silviculture and Forest Ecology of the Temperate Zones University of Göttingen Göttingen Germany; ^9^ Centre of Biodiversity and Sustainable Land Use University of Göttingen Göttingen Germany; ^10^ Vrije Universiteit Amsterdam, A‐LIFE, Section Ecology and Evolution Amsterdam The Netherlands; ^11^ Conservation and Community Ecology Group Groningen University Groningen The Netherlands; ^12^ German Centre for Integrative Biodiversity Research (iDiv) Halle‐Jena‐Leipzig Leipzig Germany; ^13^ International Institute Zittau TUD Dresden University of Technology Zittau Germany

**Keywords:** functional trait, life form, non‐native plant, resource availability, springtail, trophic guild

## Abstract

Forest ecosystem management requires the conservation of associated biodiversity. Enriching native forests with economically valuable conifer species provides economic gains and meets the increasing societal demand for timber but may threaten biodiversity. Soil sustains most of forest biodiversity, but the impact of changes in tree species composition, including native and non‐native species, on soil invertebrates remains little studied. We investigated the impact of different forest types on the taxonomic and functional composition of springtail communities (Collembola, Insecta), an abundant and diverse microarthropod group inhabiting litter and soil. Using native *Fagus sylvatica* (European beech) as reference, we compared Collembola communities with native but range‐expanding *Picea abies* (Norway spruce) and non‐native *Pseudotsuga menziesii* (Douglas fir) as well as beech–conifer mixtures. The abundance of Collembola was higher in Norway spruce than in European beech, with little difference among the other forest types. Further, the taxonomic and functional composition of Collembola was shifted to more parthenogenetic species at sandy sites, stressing the importance of regional factors such as soil type and climate in structuring Collembola communities. Collembola communities in Douglas fir were more pigmented and distributed to the surface, resulting in a lower proportion of euedaphic Collembola compared to European beech forests. In mixed forests, the impacts of Douglas fir on euedaphic Collembola were reduced, suggesting that negative effects of introduced tree species on soil animal communities might be alleviated by limiting Douglas fir to enrichment plantings only. Overall, the results indicate that vertical distribution in soil and morphological traits of Collembola help to better understand the changes in decomposer communities due to planting non‐native tree species.

## INTRODUCTION

Conserving biodiversity is essential for close‐to‐nature forest management and the long‐term provisioning of forest ecosystem functions (Bardgett & van der Putten, 2014; Binkley, 2021). Soil sustains most of forest biodiversity and carries out key ecosystem processes such as litter decomposition, carbon storage, and organic matter formation (Anthony et al., 2023; Scheu, 2005). In Central Europe, *Pseudotsuga menziesii* (Douglas fir) has become the most widely planted non‐native tree species due to its rapid growth and high‐quality wood in regions where *Picea abies* (Norway spruce), the most widespread planted native conifer species (Nicolescu et al., 2023; Schmid et al., 2014), may not cope well. However, the dominance of introduced tree species may form novel ecosystems and alter biodiversity more than planting native species outside of their natural range (Lefebvre et al., 2024; Schuldt et al., 2022). To meet the increasing demand for timber and at the same time also sustain biodiversity, mixed forests composed of native tree species and admixed non‐native tree species are increasingly established and may help in maintaining or even enhancing ecosystem functions via complementary resource use (Ammer, 2019). Yet, the impact of enrichments of forests with native tree species by non‐native tree species on soil biodiversity remains little studied (Likulunga et al., 2021; Lu & Scheu, 2021), especially with regard to the functional characteristics of soil invertebrates.

As soil detritivore invertebrates mainly live in litter and the upper soil, consuming dead organic matter and microorganisms (Potapov et al., 2022), they can shed light on processes in the opaque soil matrix as affected by changes in tree species composition (Coleman et al., 2004; Scheu, 2005; Zhou et al., 2024). Using traits that reflect habitat and resource use of soil invertebrates may allow us to better understand how tree species affect soil animal communities (McGill et al., 2006; Minor et al., 2017; Vandewalle et al., 2010). Recent studies suggested that tree species composition strongly affects aboveground herbivores and predators (Schuldt et al., 2022), whereas soil invertebrates are well buffered against changes in tree species composition due to soil organic matter stabilizing habitat conditions and the supply of food resources (Pollierer et al., 2021). By contrast, the analysis of traits of soil animals indicated that, for example, oribatid mites shift toward more surface‐living species in stands of non‐native tree species, advocating the use of functional traits in analyzing the response of belowground communities to changes in tree species (Lu et al., 2024). Overall, the use of functional traits of soil animals offers the perspective to mechanistically understand tree species—soil animal relationships, but this approach has been little used to investigate the relationship between non‐native tree species and mixed forests on soil animal communities.

Collembola are among the most diverse and abundant groups of soil mesofauna, spanning from 200 to 5000 μm in body length (Hopkin, 1997). They feed on a wide spectrum of food resources, from algae, litter, fungi, and bacteria, depending on food quality and availability, and functioning mainly as primary and secondary decomposers in soil food webs (Chahartaghi et al., 2005; Scheu & Falca, 2000). Their generalist feeding habits allow them to consume a wide spectrum of food resources (Potapov et al., 2022), thereby channeling nutrients and energy from basal resources into meso‐ and macrofauna predators (Lu, Wenglein, et al., 2025). They have evolved a range of adaptations for life in different soil layers, influencing ecosystem processes such as litter decomposition, carbon sequestration, organic matter transformation, modulating microbial communities, and root growth via trophic interactions (Potapov et al., 2020; Rusek, 1998). Based on their morphological adaptations for life in different soil layers, Collembola are grouped into euedaphic (true soil‐living), hemiedaphic (litter‐living), and epedaphic (litter surface‐living) species (Potapov et al., 2016). Compared to epedaphic and hemiedaphic species, euedaphic Collembola are characterized by the absence of body pigmentation, reduced furca length, reduction in the number of ommatidia and hair length, as well as short antennae. Collembola species have been shown to be sensitive to environmental fluctuations, for example, euedaphic species rely on relatively stable soil conditions (Holmstrup et al., 2018; Thakur et al., 2023). Furthermore, the relatively fast life history and high metabolic rate allow them to quickly respond to resource fluctuations and occupy a fast life history strategy in the soil animal economics spectrum (Lu, Pfingstl, et al., 2025).

Here, we aim to evaluate and understand changes in Collembola communities which may occur if native European beech forests are enriched with tree species planted outside of their native range. Using native *Fagus sylvatica* (European beech) forests as reference, we compared two coniferous monocultures (Norway spruce and Douglas fir) and mixtures of European beech with each conifer across a range of site conditions. Previous studies suggest that site conditions strongly modulate the effects of tree species on belowground communities (Lu & Scheu, 2021; Lwila et al., 2021) and therefore are important to consider. Douglas fir was introduced from North America more than 150 years ago, and Norway spruce naturally occurs in high mountain ranges and boreal regions but has been planted widely in lowlands (Knoke et al., 2008), providing additional comparisons for understanding the consequences of planting Douglas fir forests. We combined a taxonomic and a trait‐based approach to allow a better understanding of the mechanisms responsible for changes in Collembola communities. A range of traits was selected to reflect how Collembola interact with their environment, including trophic guild, vertical distribution in soil, and morphological traits that reflect their habitat use. Our objectives were to (1) study the structure and functions of Collembola communities in plantations of Norway spruce and Douglas fir in Central Europe, (2) evaluate the potential of admixing conifers with native tree species for conserving soil biodiversity and functions, and (3) apply a trait‐based approach combined with environmental variables to better understand the driving factors responsible for changes in soil animal communities. We hypothesized that (1) compared to European beech, Douglas fir as non‐native tree species more than Norway spruce impacts the abundance, biodiversity, species, and trait composition of Collembola; (2) mixed forests support higher abundance and biodiversity of Collembola than European beech forests; and (3) conifers, in particular Douglas fir, change the trait composition of Collembola, with these effects being mitigated in mixed forests.

## METHODS

### Study sites

**FIGURE 1:**
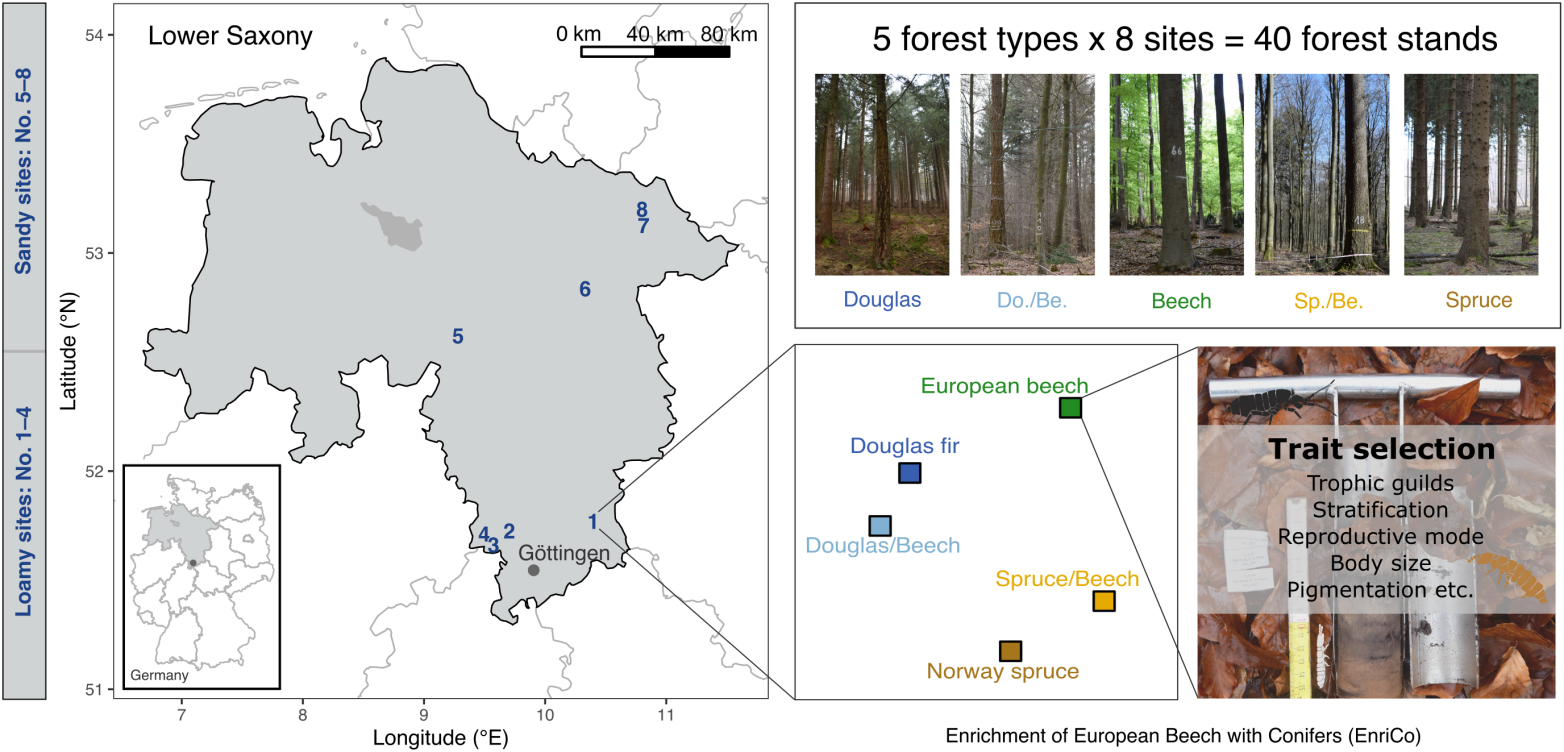
Location of the sandy and loamy study sites in Lower Saxony, Germany (Niedersachsen, Deutschland), and distribution of the five forest types investigated at each site (monocultures of European beech, Doulgas fir, and Norway spruce as well as mixed stands of European beech either with Douglas fir [Do./Be.] or Norway spruce [Sp./Be.]). Please note that the forest type location in site no. 1 is not accurate. Photo credits: Jing‐Zhong Lu. Figure modified from Lu et al. (2024).

The study included five forest types, which were replicated at each of the eight sites in Northern Germany; therefore, in total, 40 forest stands were studied (Figure 1). Each site contained stands of mature monocultures of European beech (*F. sylvatica* L.), Douglas fir (*Ps. menziesii* [Mirbel] Franco.), Norway spruce (*Pi. abies* [L.] Karst.), and two conifer–beech mixtures (Douglas fir/European beech and Norway spruce/European beech). In each stand, plots of mostly 50 × 50 m^2^ were established. The distance between plots at individual sites ranged from 76 to 4600 m. The stands were dominated by mature trees of >50 years. Focal tree species in pure stands comprised over 90% of the total basal area. Based on basal area, focal tree species in Douglas fir mixed stands consisted on average of 34% European beech and 58% Douglas fir, while in Norway spruce mixed stands, they comprised on average 56% European beech and 39% Norway spruce (Ammer et al., 2020).

The eight sites covered a range of environmental conditions, with four loamy sites in the south (51.662° N–51.770° N) stocking on parent rock of either loess‐influenced Triassic sandstone or mixtures of Paleozoic greywacke, sandstone, quartzite, and phyllite, resulting in soil types of partly podzolic Cambisols and Luvisols (Foltran et al., 2023). The mean annual precipitation at these sites is 821–1029 mm. The other four sandy sites in the north (52.621° N–53.201° N) were located on Podzols over parent material of out‐washed sand, with mean annual precipitation ranging from 672 to 746 mm. The distance between sites ranged from 5 to 190 km (Ammer et al., 2020). More details on site characteristics and soil chemical properties are given in Foltran et al. (2023).

### Environmental variables

To explain Collembola community structure, environmental variables were gathered from the same study plots by coordinated sampling as described in Lu et al. (2024). Variables were selected to represent resource availability and abiotic conditions, including root elements (C, N), litter mass, litter elements (N, P, K, Ca, Na), microbial guild composition (% bacteria and % fungi), microbial biomass, litter pH, and latitude. We primarily used variables from organic layers because the abundance and richness of Collembola decreased from litter to soil (Appendix S1: Figure S1). The number of samples taken from each plot varied from 3 to 4 and were pooled per plot, resulting in a total of 40 samples. In brief, fine roots (<2 mm) were collected from composite soil samples at 0–10 cm depth using a metal corer (Ø 8 cm). Plots were split into four quadrats, and from each, five samples were taken and pooled per quadrat. C and N concentrations were analyzed using an elemental analyzer as described in Likulunga et al. (2021). In the second campaign, composite organic layer samples from four random quadrats were taken using a metal corer (Ø 27 cm). Litter P, K, Ca, and Na concentrations were determined by inductively coupled plasma‐optical emission spectrometry (ICP‐OES; Spectro Genesis, Spectro, Kleve, Germany) after pressure digestion with nitric acid (Foltran et al., 2023). In the third sampling campaign, composite samples of three soil cores (Ø 5 cm) were taken and pooled per plot. Fatty acid markers for microbial guilds included 18:2ω6,9 for fungi, and i15:0, a15:0, i16:0, i17:0, cy17:0, cy19:0, 16:1ω7, and 18:1ω7 for bacteria (Lu & Scheu, 2021). Microbial biomass was quantified by substrate‐induced respiration (Lu & Scheu, 2021). Litter mass was measured by drying at 50°C for 48 h, and C and N contents of litter were analyzed using an elemental analyzer (Lu & Scheu, 2021). Litter pH was measured in 1 M KCl solution, a standard method that can be calibrated to water or CaCl_2_ extraction procedure (Fotyma et al., 1998). Plot latitude was included since it reflects variations in precipitation, soil type, and temperature across study sites (Ammer et al., 2020).

### Soil animal sampling

Collembola were sampled by taking soil cores using a metal corer (Ø 5 cm) between November 2017 and January 2018. Samples were taken at equidistance (>1.5 m) from trees of the same (pure stands) or different species (mixed stands). Neighborhood conifer proportions in mixed stands at a 10 m radius of our sampling points agree well with the stand‐level proportion of coniferous species (Lu et al., 2024). One soil core was taken from each plot and separated into litter, 0–5 cm and 5–10 cm depth, resulting in a total of 120 samples. Soil arthropods were extracted for each layer separately using high‐gradient heat extraction (Macfadyen, 1961). The extracted animals were collected in 50% diethylene glycol and then transferred into 70% ethanol for determination. Collembola were identified to species level using the keys of Gisin (1960), Fjellberg (1998, 2007), and Hopkin (2007).

### Functional traits

Trait values were derived from the BETSI databases (Pey et al., 2014), except for trophic guilds and vertical distribution which were measured in this study (Table 1, Appendix S1: Table S1). Traits related to feeding were represented by trophic guild and vertical distribution across the soil profile. *Trophic guild* reflects the trophic position and resource use. Based on bulk ^15^N/^14^N stable isotope ratios, three guilds were differentiated, including primary decomposers (D), secondary decomposers (I), and omnivores/predators (II) (Chahartaghi et al., 2005). Primary decomposers predominantly feed on litter, whereas secondary decomposers are mainly mycophagous, and omnivores/predators occupy high trophic positions due to incorporation of old organic matter or microfauna (Li et al., 2022). *Vertical distribution* across the soil profile reflects habitat use and vertical niche differentiation (Ellers et al., 2018; Potapov et al., 2016). Vertical distribution of each species was estimated by abundance‐weighted mean depth of Collembola species based on the sampled layers (litter, 0–5 cm, and 5–10 cm) across all plots. Higher values reflect more surface living than soil‐dwelling. Although trophic position is related to life forms of Collembola, the high variability of trophic positions within life forms supports the inclusion of both as functional traits (Potapov et al., 2016).

**TABLE 1:** List of Collembola traits used in this study and their assumed function.

Traits	Unit	Functions	Source
Trophic guilds (^15^N/^14^N)	Categorical (D, I, and II)	Trophic position and resource use	This study
Vertical distribution across soil	Numeric	Vertical niche differentiation and habitat use	This study
Reproductive mode	Categorical (sex, parthenogenesis)	Sexual species reflecting resource limitation and/or abiotic harshness	BETSI
Body size	Numeric	Energy use and mobility	BETSI
Antenna/body length ratio	Numeric	Adaptation to soil surface, movement	BETSI
Furcal size	Numeric	Jumping ability and escaping predators	BETSI
Pigmentation	Numeric	Adaptation to surface living and camouflage	BETSI
No. ocelli	Numeric	Adaptation to surface living	BETSI

Life history traits were represented by reproductive mode. *Reproductive mode* included sexual and parthenogenetic reproduction, indicated by the presence of males in the population recorded in the literature (e.g., Fjellberg, 1998, 2007). The prevalence of sexual reproduction reflects resource limitation or patchiness, with parthenogenetic species thriving in habitats with abundant and easily accessible resources supporting large populations (Maraun et al., 2019; Scheu & Drossel, 2007).

Morphological traits were represented by five traits: (1) *Body size* is a master trait reflecting environmental tolerance (Van Dooremalen et al., 2013), energy use, and mobility (Peters, 1983; Potapov et al., 2019); large body size enables individuals to be more efficient in extracting energy from low‐quality food (Brown & Maurer, 1986) but restricts access to resources deeper in soil. (2) *Antenna/body length ratio* is the relative length of antenna reflecting surface versus soil living. (3) *Furcal size* reflects the ability to jump and escape predators. (4) *Pigmentation* provides camouflage and serves to absorb radiation (Rapoport, 1969). (5) *Number of ocelli* reflects adaptation to surface living.

### Calculation of functional diversity and community metabolism

All calculations and analyses were performed in R 4.3.0 (R Core Team, 2023). Distance‐based functional diversity indices were calculated using the FD package (Laliberté et al., 2014). Functional evenness and divergence were derived in a multidimensional trait‐space taking population abundance into account (Villéger et al., 2008). Functional evenness describes the regularity of the distribution of species within the functional space defined by multiple traits, while functional divergence quantifies the degree of trait dispersion from the centroid in functional space. In addition to functional diversity, total biomass and metabolic rate were computed. Fresh body mass of Collembola was calculated from body length and width based on the power equations for Arthopleona DW = *a* × *L* ^*b*^ and Symphypleona DW = *a* × (0.83 × *L*)^*b*^, with DW the dry weight (in micrograms), *L* the body length (in millimeters), and *a* and *b* coefficients of 5.6 and 2.693 for Arthropleona and 57.8 and 2.954 for Symphypleona, respectively (Petersen, 1975). Fresh mass (in micrograms) was estimated using FM = *c* × DW^*d*^, with *c* and *d* being coefficients of 4.08 and 1.02, respectively (Mercer et al., 2001). Individual metabolic rate of Collembola was quantified following the equation *I* = *i*_0_ *M* ^*a*^ e^−*E*/*kT*^, with *I* the metabolic rate (in joules per hour), *i*_0_ a normalization factor (3,477,995,118), *M* for fresh mass in milligrams, *a* the allometric exponent (0.759), *E* the activation energy (0.657 eV), *k* the Boltzmann constant (8.62 × 10^−5^ eV K^−1^), and *T* the temperature in Kelvin (Ehnes et al., 2011). The mean annual temperature at our study plots was taken from Ammer et al. (2020). Community metabolism was calculated as the sum of individual metabolic rates. We used community metabolism as this metric is more universal across the size spectrum of organisms than biomass or abundance (Brown et al., 2004; Potapov et al., 2021) and can serve as a proxy for the total activity or consumption rate (Barnes et al., 2018; Potapov et al., 2023).

### Statistical analysis

Linear mixed‐effects models (LMMs) were used to analyze the effects of forest type (European beech, Douglas fir, Norway spruce, Douglas fir/European beech, Norway spruce/European beech), site condition (Loamy and Sandy sites), and their interactions on attributes of Collembola communities. Response variables included abundance, biomass, community metabolism, species richness, functional evenness and divergence, guild compositions, community weighted mean (CWM) trait value (pigmentations, vertical stratification), and life form compositions. The random effect included the eight sites to account for nesting of forest types within each site.

Principal components analysis (PCA) was used to compare species and trait composition of Collembola. Trait composition of communities was calculated using CWM trait values by the abundance of each species (Appendix S1: Table S1). Numeric trait variables were standardized to range between 0 and 1, and dummy variables were used for categorical traits (0 or 1). Standardized environmental variables (*r* < 0.8) that predicted Collembola community structure were fitted in the PCA using the env.fit function. Furthermore, permutational multivariate analyses of variance (PERMANOVA) were conducted to inspect the effects of forest type, site condition, and their interactions on Collembola taxonomic and trait composition. Multivariate homogeneity of dispersion (beta‐diversity) was also assessed for forest types using “betadisper” (Anderson et al., 2006). Only species that occurred in more than one plot were included in the multivariate analysis of species composition.

For univariate analysis, data were log or square root transformed to meet model assumptions. The package “nlme” was used for LMMs (Pinheiro et al., 2019). The emmeans function was used for estimated marginal means, and the contrast function to test the significance among forest types using European beech as reference (Lenth, 2019). PERMANOVA were based on Bray–Curtis dissimilarities; “vegan” was used for PERMANOVA (“adonis2”) analysis (Oksanen et al., 2019). Residual plots were inspected visually to examine normality and homoscedasticity assumptions.

## RESULTS

**FIGURE 2:**
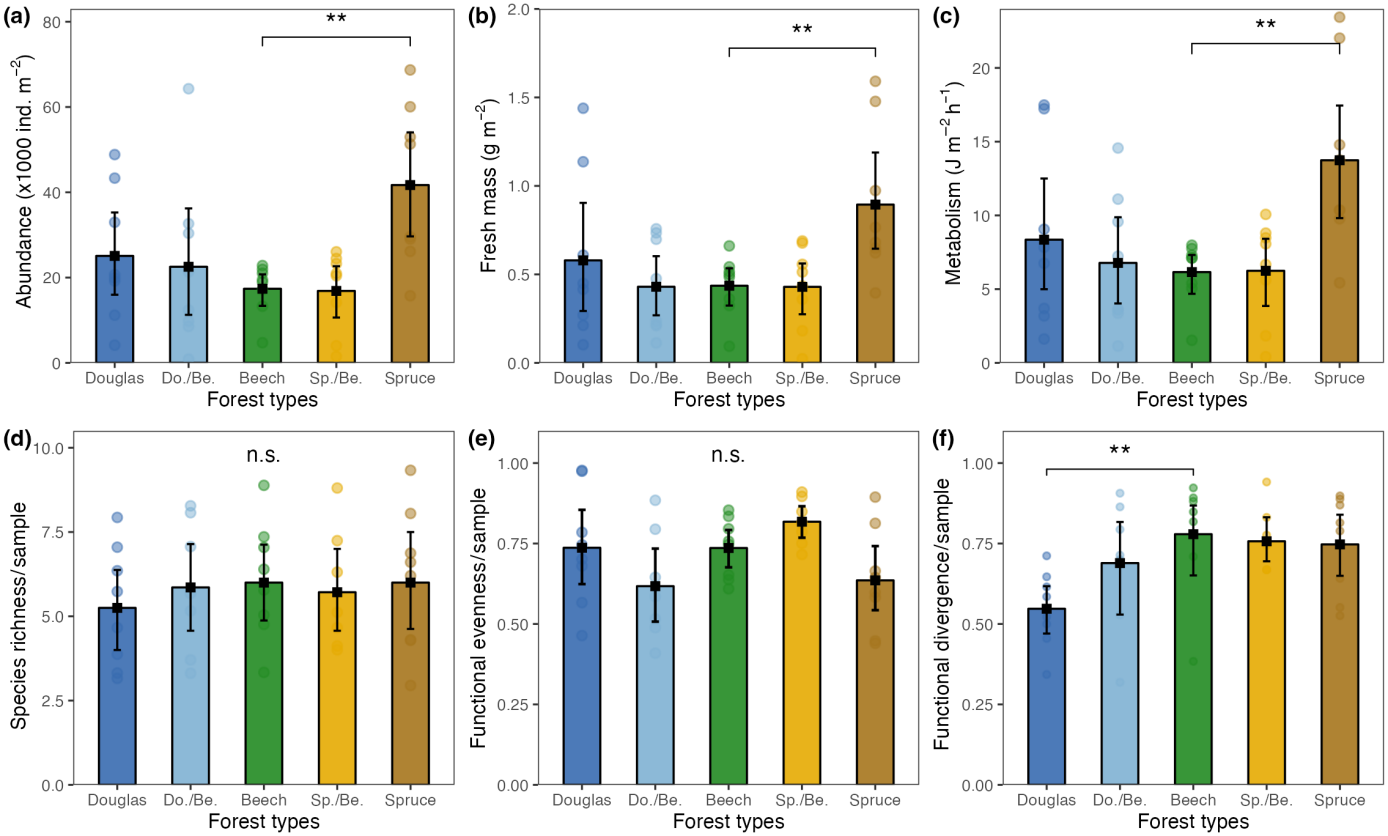
Abundance and diversity metrics of Collembola in five forest types: Douglas fir (Douglas), European beech (Beech), Norway spruce (Spruce), and two conifer–beech mixtures: Douglas and Beech (Do./Be.) and Spruce and Beech (Sp./Be.). (a) Total number of individuals per square meter, (b) total fresh mass per square meter, (c) community metabolism per square meter per hour, (d) number of species per soil core, (e) functional evenness, and (f) functional divergence per sample. Means and 95% confidence levels; significant differences between European beech and other forest types are denoted by asterisks at the line linking the respective forest types (**0.001 ≤ *p* < 0.01, “n.s.” *p* > 0.05).

**FIGURE 3:**
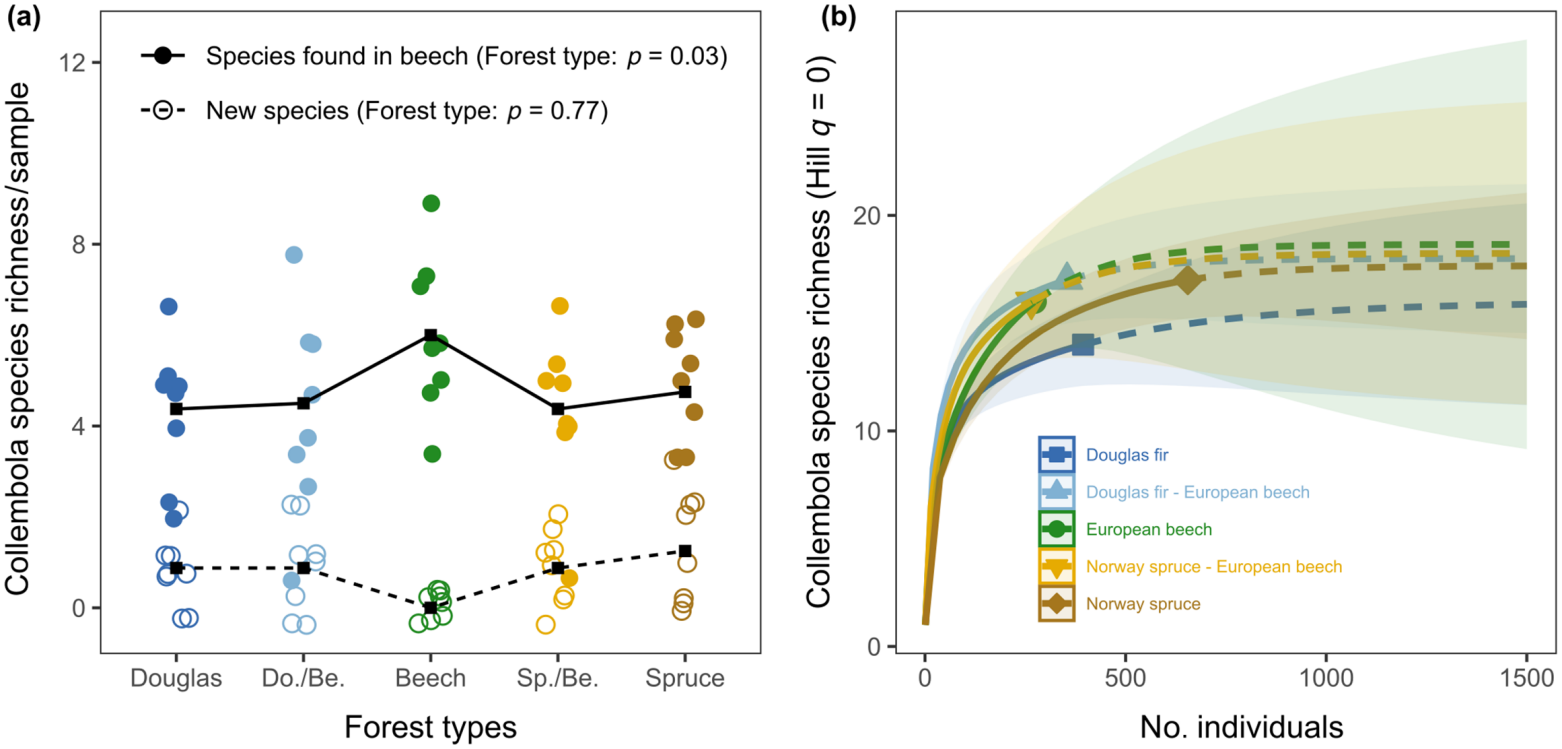
Collembola species turnover with forest management and gamma diversity. (a) Changes in the number of species found in beech forests or new species (that are not found in beech); mean values are connected by lines to improve visibility. (b) Rarefaction and extrapolation (solid and dashed lines, respectively) of beta diversity (species turnover with increasing number of individuals) and gamma diversity in five forest types including monoculture of Douglas fir, European beech, Norway spruce, and mixed stands of European beech with Douglas fir (Do./Be.) or Norway spruce (Be./Sp.). Bands in (b) are 95% CI.

In total, 26 species were identified from 1941 individuals of Collembola. The most abundant species were *Folsomia quandrioculata*, *Parisotoma notabilis*, *Isotomiella minor*, *Protaphorura armata*, and *Folsomia manolachei*, accounting for 82.3% of all individuals. In European beech forests, the mean abundance of Collembola was 17.3 ± 5.09 × 10^3^ ind. m^−2^, the mean fresh biomass was 414.6 ± 90.73 mg m^−2^, and the mean community metabolism was 5.9 ± 1.37 J m^−2^ h^−1^ (Appendix S1: Table S2; Figure 2). In Norway spruce monoculture, the abundance, biomass, and community metabolism of Collembola were, on average, 2.2 times higher than in European beech forests (Figure 2a–c). Further, functional divergence was significantly lower in Douglas fir than in European beech forests (−0.23; Figure 2f). Species richness per sample in European beech forests was about 1.5 times higher than in coniferous and mixed forests (Figure 3a), but the overall species richness, functional evenness, and gamma diversity of Collembola did not significantly differ among forest types (Figures 2d,e and 3b).

**FIGURE 4:**
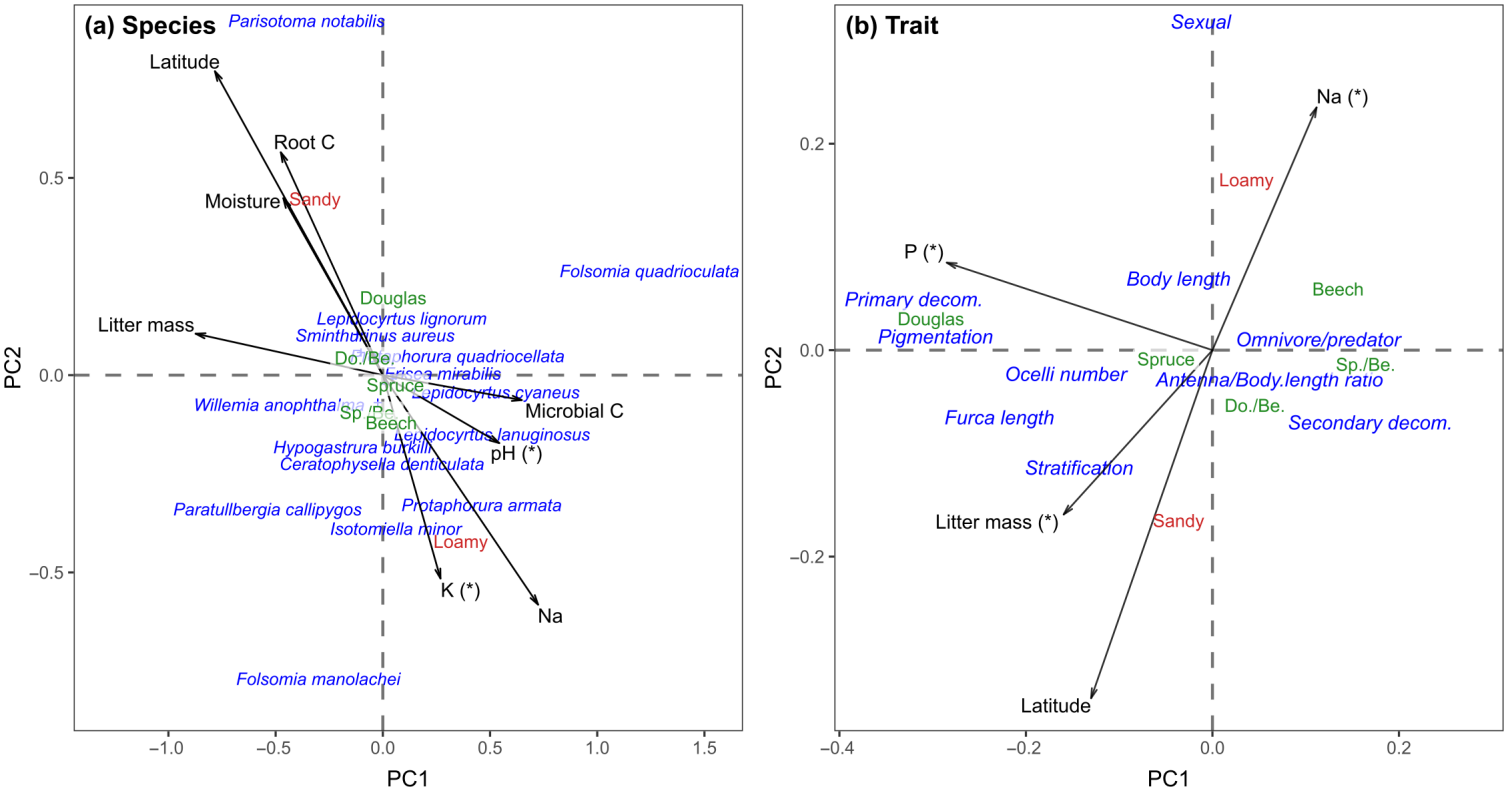
Principal components analysis (PCA) of (a) species and (b) community traits of Collembola in five forest types, including Douglas fir, Douglas fir/European beech (Do./Be.), European beech, Norway spruce/European beech (Sp./Be.), and Norway spruce at loamy (southern) and sandy (northern) sites. Significant environmental variables are indicated by arrows in black (*p* < 0.05; (*) *p* < 0.1).

The community composition of Collembola was most strongly affected by site conditions (PERMANOVA, *F*_1,30_ = 3.81, *p* = 0.001). As indicated by PCA, the taxonomic composition of Collembola was affected by root carbon content, litter moisture content, litter mass, as well as Na concentration and microbial biomass (Figure 4a). By contrast, the trait composition of Collembola did not significantly vary with forest type or site conditions (*p* > 0.14 for both) but with plot latitude. Higher latitude was associated with more parthenogenetically reproducing species, higher root carbon concentration, and litter moisture content, but this also (negatively) correlated with low litter Na concentration (Figure 4b). Further, the first PCA axis mainly separated surface‐living Collembola with strong pigmentation, long furca, and living as primary decomposers from soil‐living ones predominantly living as secondary decomposers; this axis correlated negatively with litter P concentration (although only marginally significant).

**FIGURE 5:**
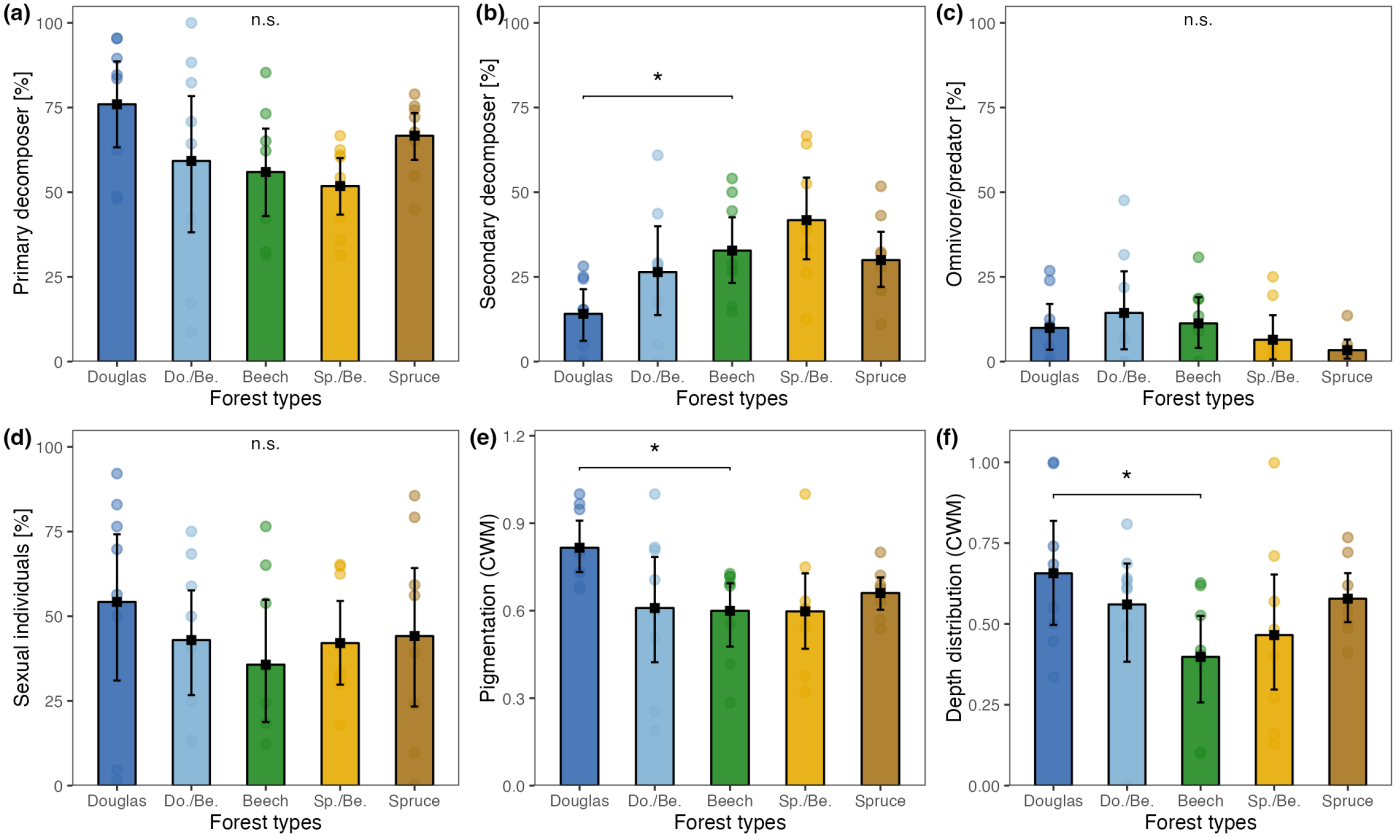
Comparison of the guild composition of Collembola across five forest types, including European beech (Beech), Douglas fir (Douglas), Norway spruce (Spruce), and two conifer–beech mixtures (Do./Be. and Sp./Be.). The proportion of (a) primary decomposer, (b) secondary decomposer, (c) omnivore/predator individuals, (d) mode of reproduction, (e) pigmentation (community weighed mean, CWM), and (f) the vertical distribution across the soil profile (CMW, scaled). Means and 95% confidence levels; significant differences between European beech and other forest types are denoted by asterisks at the line linking the respective forest types (*0.01 ≤ *p* < 0.05, “n.s.” *p* > 0.05).

**FIGURE 6:**
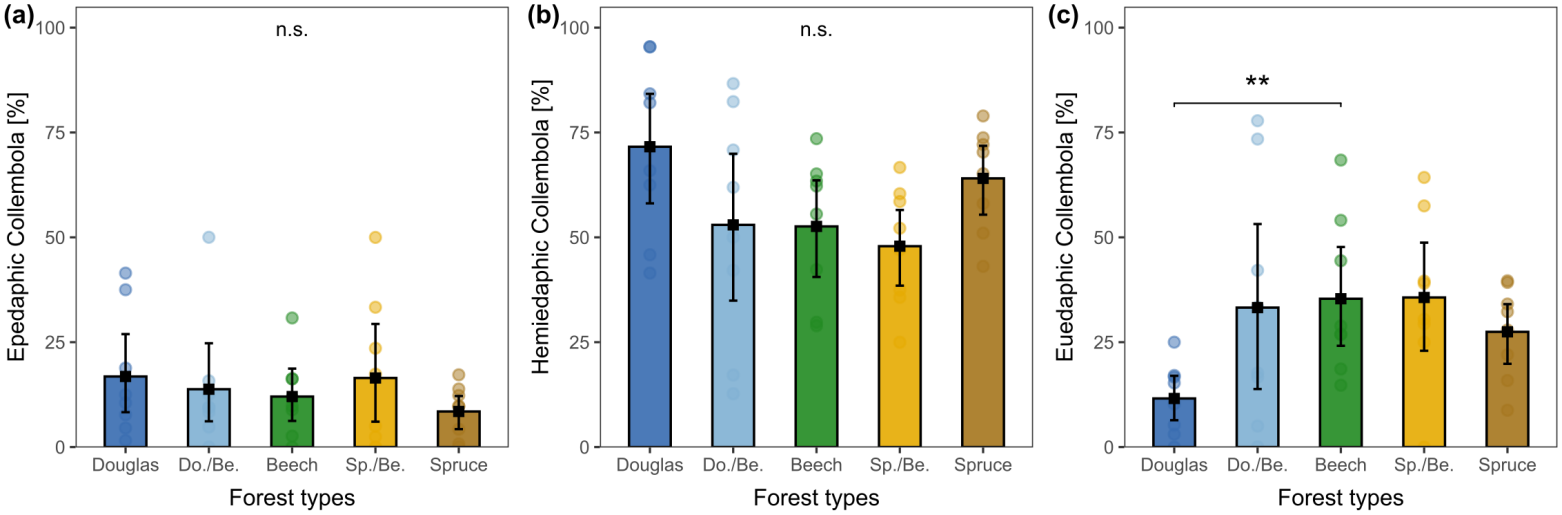
Variations in the relative abundance of life forms of Collembola with forest type: (a) epedaphic, (b) hemiedaphic, and (c) euedaphic Collembola. Forest types included European beech (Beech), Douglas fir (Douglas), Norway spruce (Spruce), and two conifer–beech mixtures (Do./Be. and Sp./Be.). Means and 95% confidence levels; significant differences between European beech and other forest types are denoted by asterisks at the line linking the respective forest types (**0.001 ≤ *p* < 0.01, “n.s.” *p* > 0.05).

Functional groups of Collembola significantly differed among forest types. The proportion of secondary decomposers (overall mean 32.8% ± 15.0%) was 57% lower in Douglas fir than in European beech forests (Appendix S1: Table S3; Figure 5b). Further, compared to European beech forests (as well as other forest types), Douglas fir forests were colonized by stronger pigmented Collembola (+0.22 CWM pigmentation) and by Collembola living closer to the soil surface (+0.14 CWM vertical distribution; Figure 5e,f). In addition, compared to European beech, the relative abundance of euedaphic Collembola in Douglas fir forests was strongly reduced (Figure 6c), which was mainly due to increased relative abundance of hemiedaphic species in Douglas fir forests (Figure 6b). Generally, the biomass, community metabolism, and species richness of Collembola at loamy sites exceeded that at sandy sites by factors of 1.96, 1.82, and 1.49, respectively, and the proportion of sexually reproducing species at loamy sites exceeded that at sandy sites by a factor of 2.45 (Appendix S1: Table S4).

## DISCUSSION

Introduced plants and their impact on ecosystems have become a global issue and may threaten local biodiversity (Vitousek et al., 1997). Using native European beech as reference, we evaluated the impact of introduced Douglas fir and native but range‐expanding Norway spruce on soil Collembola communities, combining both taxonomy‐ and trait‐based approaches. We found that the abundance and local species number are, in general, not negatively affected by Douglas fir, but the functional composition of Collembola was shifted toward more surface‐living and pigmented species, resulting in lower abundance of euedaphic Collembola in Douglas fir compared to European beech forests. Our findings stress the necessity to study the functional structure of soil biodiversity to complement taxonomic composition and to better understand tree species – decomposer relationships. The negative impacts of Douglas fir on euedaphic Collembola were mitigated in mixed forests of European beech and Douglas fir, suggesting that tree mixtures hold promise for maintaining soil functions when planting non‐native Douglas fir.

### Collembola community structure as affected by Douglas fir and Norway spruce

In contrast to our first hypothesis, species richness of Collembola did not significantly differ between forest types, suggesting that Collembola diversity is resistant to changes in tree species composition. The abundance, biomass, and community metabolism of Collembola in Douglas‐fir were similar to those in European beech forests but were much higher in Norway spruce forests, suggesting that Norway spruce forests provide more abundant food and habitat for soil Collembola than European beech forests. Both Norway spruce and Douglas fir are coniferous tree species, but their effects on soil Collembola differed. This contrasts with the response of soil Oribatida from the same sampling sites (Lu et al., 2024) and suggests that Collembola benefit more from thick organic layers and potentially also loose packing of needles in Norway spruce forests (Foltran et al., 2023; Fujii et al., 2020) than Oribatida. In contrast to other groups, such as ground arthropods and canopy birds (Schuldt et al., 2022; Wildermuth et al., 2023), the overall impact of Douglas fir on the abundance and diversity of Collembola was relatively weak, suggesting that Douglas fir generally provides ample food and habitat for Collembola. Forest soils are heterogeneous and rich in organic matter, providing a wide range of resources for decomposer animals, thereby likely buffering against environmental fluctuations and changes (Fujii et al., 2020; Pollierer et al., 2021). Further, soil microarthropods including Collembola are food generalists, and this likely contributes to their resistance to changes in environmental conditions such as changes in tree species composition and climate (Bonfanti et al., 2022; Susanti et al., 2021).

Compared to beech, the proportion of secondary decomposers (Chahartaghi et al., 2005) was reduced in Douglas fir, suggesting that Douglas fir limits the population size of more surface‐living, microbivorous Collembola but not that of primary decomposers. Douglas fir shifts the functional composition of Collembola toward living in litter, further supporting our conclusion of ample litter‐based resources but reduced soil‐based resources in Douglas fir. Indeed, Douglas fir significantly reduced the proportion of euedaphic Collembola compared to European beech forests, stressing the importance of vertical stratification in understanding the response of soil animal communities to changes in tree species composition. Supporting these results, euedaphic Collembola have been found to be most responsive to forest conversion (Chauvat et al., 2011). Changes in trait composition of Collembola also highlighted the importance of evaluating soil biodiversity across soil depths (Ellers et al., 2018; Krab et al., 2010), presumably reflecting the two resource channels via litter and root‐derived resources.

Previous studies suggested euedaphic Collembola such as *Protaphorura armata* to be associated with roots (Li et al., 2020; Remén et al., 2008; Salamon et al., 2004), and therefore lower densities in Douglas fir forests likely indicates shortage of root‐derived resources. Further, Douglas fir was found to negatively impact soil microbial biomass (Lu & Scheu, 2021), the relative abundance of symbiotrophic fungi (Likulunga et al., 2021), and fine root biomass compared to European beech forests (Lwila et al., 2021). Similar to Collembola, Douglas fir also changed the vertical distribution of soil Oribatida at our study sites toward more surface living (Lu et al., 2024). These results imply that although, based on species composition, Collembola are resistant to changes in tree species composition, changes in soil resources result in shifts in their functional composition. These findings support that Collembola traits and life forms are highly responsive to environmental change (Holmstrup et al., 2018) and indicate that, when analyzed together with trophic guilds, they allow to identify the responsible mechanisms.

### Tree mixtures mitigate the impact of Douglas fir on euedaphic Collembola

The lower abundance of euedaphic Collembola in Douglas fir but not in Norway spruce in comparison with beech suggests that Douglas fir and Norway spruce differently affected soil Collembola communities, arguing for caution when replacing Norway spruce forests with Douglas fir in monocultures as this may change belowground processes and functions. In contrast to our second hypothesis, we did not find higher biodiversity of soil Collembola communities in mixed forests, and the guild composition of Collembola was generally in between the respective monocultures, suggesting that mixed forests may not increase soil biodiversity but mitigate the potential negative impacts of Douglas fir. This is consistent with the findings for not only other groups of soil biota, such as soil microbial communities, oribatid mites, and macrofauna (Lu et al., 2024; Lu & Scheu, 2021; Wenglein et al., 2024), but also canopy arthropods and birds (Schuldt et al., 2022; Wildermuth et al., 2023). Trait composition of Collembola was most strongly impacted by Douglas fir, and this was associated with a lower proportion of euedaphic Collembola in Douglas fir; the impacts were alleviated in mixed forests supporting our third hypothesis that mixed Douglas fir forests are beneficial for maintaining trait composition of Collembola similar to native forests in Central Europe. The measured environmental variables poorly correlated with the community structure of Collembola, supporting earlier assumptions that environmental factors measured at the bulk soil level may not adequately reflect the factors structuring Collembola communities (Ferlian et al., 2015). Earlier studies indicated that Douglas fir only affects microbial biomass and community structure at sandy sites (Lu & Scheu, 2021). As microbial guild compositions and microbial biomass measured in bulk soil are poor predictors of Collembola community structure, it is not surprising that the response of Collembola to forest types did not follow that of microorganisms and did not vary with site conditions. However, site conditions are key drivers for the community structure of soil Collembola, supporting that regional factors are more influential than forest types in structuring the community structure of Collembola (Erdmann et al., 2012; Pollierer & Scheu, 2017). Site conditions, such as soil nutrient concentrations and litter moisture content, intercorrelate with other environmental variables such as annual mean precipitation, and therefore, the drivers of the variation in microarthropod community structure with site conditions are difficult to disentangle. At the time of sampling (November–January), litter moisture content at sandy sites was higher than at loamy sites, presumably reflecting increased precipitation in autumn, in particular at sandy sites, although mean annual precipitation was lower at sandy than at loamy sites (Glatthorn et al., 2023). Further, it is interesting to note that sodium concentrations were higher at loamy sites than at sandy sites and sodium concentration was identified as a significant variable driving Collembola community structure. This likely reflects that sodium may be more limited at our sandy sites, influencing soil food webs (Kaspari et al., 2014; Welti et al., 2019).

In conclusion, functional traits of Collembola shed light on the mechanisms driving changes in Collembola community structure and highlighted that vertical distribution across the soil profile and trophic interactions are key to understanding the impact of changes in tree species composition on soil biodiversity. Soil functional ecology is thriving thanks to the advancement of databases and analytical approaches that document what species do and how they respond to environmental changes (Ellers et al., 2018; Pey et al., 2014). Trait‐based analyses integrate species‐level community analyses with morphology and feeding ecology, allowing one to go beyond simple biodiversity measures by uncovering mechanisms responsible for the response of soil biota to environmental changes, such as planting non‐native tree species. In the face of global change, sustainable forest management meeting both the increasing demand for forest products and the growing awareness of the necessity to conserve soil biodiversity is urgently needed. The admixture of target tree species in mixed stands help mitigate potential negative impacts of monocultures and introduced tree species on soil biodiversity, thereby contributing to reaching the goal of close‐to‐nature forest management.

## AUTHOR CONTRIBUTIONS

Jing‐Zhong Lu and Stefan Scheu designed the study. Jing‐Zhong Lu, Junbo Yang, Anton Potapov, Andrea Polle, Matty Berg, Estela Foltran, Carmen Alicia Rivera Pérez, Jonas Glatthorn, Christian Bluhm, and Stefan Scheu contributed the data. Jing‐Zhong Lu analyzed the data and wrote the first draft of the manuscript. All authors contributed substantially to the interpretation and revisions.

## CONFLICT OF INTEREST STATEMENT

The authors declare no conflicts of interest.

## Supporting information


Appendix S1:

## Data Availability

Data (Lu & Scheu, 2022) are available in PANGAEA at https://doi.org/10.1594/PANGAEA.944669.
