# Supplementary material for: Mixed forests with native species mitigate impacts of introduced Douglas fir on soil decomposers (Collembola)
Source: Ecol Appl. 2025 May 4;35(3):e70034. doi: 10.1002/eap.70034 (PMC12050398; doi:10.1002/eap.70034)
Supplement: Supplementary file 1 — Appendix S1: [file EAP-35-e70034-s001.pdf]

Appendix S1

Supplementary materials for

Mixed forests with native species mitigate impacts of introduced Douglas fir on soil-living decomposers (Collembola)

Authors: Jing-Zhong Lu, Junbo Yang, Christian Bluhm, Estela Foltran, Carmen Alicia Rivera Pérez, Jonas Glatthorn, Christian Ammer, Norbert Lamersdorf, Andrea Polle, Matty Berg, Anton M. Potapov, Stefan Scheu

Table S1. List of species and trait values of Collembola species. Note that body length in the table is the maximum body length and was corrected for biomass estimation using the power equation L [mm] = e × (Body length; mm)^f, with coefficients e and f estimated to be 0.7447624 and 0.62406 using individuals from this study. Life forms were assigned based on Potapov et al. (2016). Trophic positions include primary decomposer (D), and secondary decomposer (I) and omnivore/predator (II; sensu Chahartaghi et al. 2005).

Species	family	Antenna/ Body Ratio	Body length mm	Pigment scaled	Ocelli numbe r	Fu rca	Reprod uctive mode	Stratifi cation scaled	Trop hic posit ion	morphgroups
Ceratophysella denticulata	Hypogastruridae	0.11	1.7	1	8	1	1	0.09	II	epedaphic
Desoria violacea	Isotomidae	0.25	2.5	1	8	2	1	0.93	D	epedaphic
Dicyrtomina ornata	Dicyrtomidae	0.74	2.5	1	8	2	1	0.80	D	atmobiotic
Entomobrya nivalis	Entomobryidae	0.52	2.2	1	8	2	1	0.73	I	atmobiotic
Folsomia manolachei	Isotomidae	0.17	0.9	1	2	2	1	0.69	D	hemiedaphic
Folsomia quadrioculata	Isotomidae	0.17	1.7	0.8	2	2	1	0.68	D	hemiedaphic
Frisea mirabilis	Neanuridae	0.13	1.6	1	8	1	1	0.41	II	epedaphic
Hypogastrura burkilli	Hypogastruridae	0.13	2.8	1	8	1	1	0.67	I	epedaphic
Isotomiella minor	Isotomidae	0.27	1.0	0	0	2	0	0.65	I	euedaphic
Lepidocyrtus lanuginosus	Entomobryidae	0.38	2.0	0.25	8	2	1	0.74	I	epedaphic
Lepidocyrtus cyaneus	Entomobryidae	0.38	1.5	1	8	2	1	0.87	D	epedaphic
Lepidocyrtus lignorum	Entomobryidae	0.38	1.7	0.80	8	2	1	1.00	I	epedaphic
Lepidocyrtus violaceus	Entomobryidae	0.38	1.5	1	8	2	1	0.87	I	epedaphic
Mesaphorura macrochaeta	Tullbergiidae	0.09	0.7	0	0	0	0.25	0.85	II	euedaphic
Neanura muscorum	Neanuridae	0.16	3.3	1	3	0	0.29	0.80	II	hemiedaphic
Orchesella bifasciata	Entomobryidae	0.74	2.8	1	8	2	1	0.87	D	atmobiotic
Paratullbergia callipygos	Tullbergiidae	0.17	1.0	0	0	0	0.33	0.18	II	euedaphic
Parisotoma notabilis	Isotomidae	0.28	1.1	1	4	2	0.4	0.89	D	hemiedaphic
Pogonognathellus flavescens	Tomoceridae	1.25	5.7	1	8	1	1	0.54	D	atmobiotic
Protaphorura armata	Onychiuridae	0.11	2.1	0	0	0	1	0.00	I	euedaphic
Protaphorura quadriocellata	Onychiuridae	0.11	2.1	0	0	0	1	0.59	II	euedaphic
Pseudosinella alba	Entomobryidae	0.34	1.0	0.17	2	2	1	0.80	II	hemiedaphic
Sminthurinus aureus	Katiannidae	0.44	0.9	1	8	2	1	0.80	D	epedaphic
Willemia anophthalma	Hypogastruidae	0.1	0.9	0	0	0	0	0.87	II	euedaphic
Willemia denisi	Hypogastruidae	0.11	0.8	0	0	0	0	0.80	II	euedaphic
Xenyllodes armata	Odontellidae	0.07	0.8	1	5	1	0	0.61	I	hemiedaphic

Table S2. Estimated marginal means based linear mixed-effects models for the effect of forest type (European beech, Douglas fir, Norway spruce and two conifer- beech mixtures) on total abundance, fresh mass, community metabolism, species richness, functional evenness (Feve) and divergence (Fdvi) of Collembola per sample (area of 19.6 cm^2). CL refers to 95% confidence level estimated based on the “emmeans” package. P-values derived from contrast against European beech. Significant effects are given in bold ($p \leq 0.05$).

Forest type	Mean	Standard Error	Lower CL	Upper CL	Unit	Response variable	P-value (Contrast vs. beech)
Douglas	49.250	10.048	24.663	73.837	count	Abundance	0.281
Douglas-Beech	44.250	10.048	19.663	68.837	count	Abundance	0.467
Beech	34.125	10.048	9.538	58.712	count	Abundance	-
Spruce-Beech	33.125	10.048	8.538	57.712	count	Abundance	0.942
Spruce	81.875	10.048	57.288	106.462	count	Abundance	**0.002**
Douglas	983.183	195.773	562.493	1520.574	µg	Fresh body mass	0.489
Douglas-Beech	766.156	172.820	401.631	1247.384	µg	Fresh body mass	0.834
Beech	814.103	178.146	436.547	1308.362	µg	Fresh body mass	-
Spruce-Beech	752.888	171.318	392.041	1230.439	µg	Fresh body mass	0.788
Spruce	1673.974	255.453	1107.255	2357.396	µg	Fresh body mass	**0.005**
Douglas	14.463	3.008	8.040	22.759	mJ h-1	Community metabolism	0.441
Douglas-Beech	11.834	2.721	6.113	19.427	mJ h-1	Community metabolism	0.950
Beech	11.614	2.695	5.955	19.145	mJ h-1	Community metabolism	-
Spruce-Beech	10.891	2.610	5.441	18.213	mJ h-1	Community metabolism	0.833
Spruce	25.763	4.014	16.877	36.522	mJ h-1	Community metabolism	**0.003**
Douglas	5.250	0.569	3.858	6.642	count	Species richness	0.284
Douglas-Beech	5.727	0.607	4.242	7.213	count	Species richness	0.707
Beech	6.000	0.569	4.608	7.392	count	Species richness	-
Spruce-Beech	5.448	0.607	3.962	6.934	count	Species richness	0.449
Spruce	6.000	0.569	4.608	7.392	count	Species richness	1.000
Douglas	0.737	0.050	0.616	0.858	none	Feve	0.990
Douglas-Beech	0.605	0.053	0.475	0.735	none	Feve	0.060
Beech	0.736	0.050	0.615	0.857	none	Feve	-
Spruce-Beech	0.829	0.053	0.699	0.959	none	Feve	0.172
Spruce	0.636	0.050	0.514	0.757	none	Feve	0.126
Douglas	0.547	0.056	0.410	0.685	none	Fdiv	**0.005**
Douglas-Beech	0.701	0.061	0.553	0.849	none	Fdiv	0.323
Beech	0.779	0.056	0.641	0.916	none	Fdiv	-
Spruce-Beech	0.757	0.061	0.609	0.905	none	Fdiv	0.778
Spruce	0.747	0.056	0.610	0.885	none	Fdiv	0.672

Table S3. Estimated marginal means based linear mixed-effects models on the effect of forest type (European beech, Douglas fir, Norway spruce and two conifer- beech mixtures) on primary and secondary decomposer, and omnivore/predator Collembola (sensu Chahartaghi et al. 2005), as well as community weighed mean of reproductive mode, pigmentation and depth distribution (stratification, scaled). CL refers to 95% confidence level estimated based on the "emmeans" package. P values derived from contrast against European beech. Significant effects are given in bold (p ≤ 0.05).

Forest type	Means	Standard error	Lower CL	Upper CL	Unit	Response variable	P-value (Contrast vs. beech)
Douglas	74.706	9.750	52.753	100.468	proportion	Primary decomposer	0.093
Douglas-Beech	53.467	8.249	35.188	75.555	proportion	Primary decomposer	0.929
Beech	54.427	8.322	35.968	76.695	proportion	Primary decomposer	-
Spruce-Beech	50.981	8.055	33.177	72.594	proportion	Primary decomposer	0.746
Spruce	66.195	9.178	45.642	90.558	proportion	Primary decomposer	0.305
Douglas	9.941	4.255	2.256	23.078	proportion	Secondary decomposer	**0.015**
Douglas-Beech	21.075	6.195	8.642	38.959	proportion	Secondary decomposer	0.296
Beech	31.224	7.541	15.499	52.401	proportion	Secondary decomposer	-
Spruce-Beech	39.664	8.499	21.594	63.186	proportion	Secondary decomposer	0.454
Spruce	28.664	7.225	13.711	49.069	proportion	Secondary decomposer	0.804
Douglas	6.294	3.859	0.393	19.278	proportion	Omnivore/predator	0.929
Douglas-Beech	7.876	4.317	0.855	21.980	proportion	Omnivore/predator	0.844
Beech	6.766	4.001	0.518	20.098	proportion	Omnivore/predator	-
Spruce-Beech	2.665	2.511	0.000	12.350	proportion	Omnivore/predator	0.356
Spruce	1.852	2.093	0.000	10.514	proportion	Omnivore/predator	0.240
Douglas	45.153	10.771	22.643	75.353	proportion	Reproductive mode	0.217
Douglas-Beech	39.444	10.067	18.657	67.922	proportion	Reproductive mode	0.398
Beech	28.633	8.577	11.491	53.466	proportion	Reproductive mode	-
Spruce-Beech	40.200	10.163	19.178	68.914	proportion	Reproductive mode	0.368
Spruce	35.445	9.543	15.940	62.641	proportion	Reproductive mode	0.582
Douglas	0.816	0.070	0.646	0.986	standardized CWM	Pigmentation	**0.022**
Douglas-Beech	0.609	0.070	0.439	0.779	standardized CWM	Pigmentation	0.914
Beech	0.599	0.070	0.429	0.770	standardized CWM	Pigmentation	-
Spruce-Beech	0.598	0.070	0.427	0.768	standardized CWM	Pigmentation	0.983
Spruce	0.661	0.070	0.490	0.831	standardized CWM	Pigmentation	0.494
Douglas	0.657	0.082	0.457	0.857	scaled CWM	Stratification	**0.035**
Douglas-Beech	0.560	0.082	0.360	0.760	scaled CWM	Stratification	0.174
Beech	0.398	0.082	0.198	0.598	scaled CWM	Stratification	-
Spruce-Beech	0.455	0.082	0.266	0.665	scaled CWM	Stratification	0.565
Spruce	0.578	0.082	0.378	0.778	scaled CWM	Stratification	0.133

Table S4. Estimated marginal means based linear mixed-effect models on the effect of site conditions (loamy sites, sandy sites) on total abundance, fresh mass, community metabolism, species richness, functional evenness (FEve) and divergence (FDvi), proportion of primary decomposer, secondary decomposer and omnivore/predator (sensu Chahartaghi et al. 2005), sexual species and community weighed means (CWM) of pigmentation and depth distribution (stratification) as well as proportion of life forms of Collembola per sample (area of 19.6 cm^2). CL refers to 95% confidence level estimated based on the "emmeans" package.

Site condition	Means	Standard error	Lower CL	Upper CL	Unit	Response variable	P-value (Contrast vs. Sandy)
Loamy	59.25	7.18	42.27	76.23	Count	Abundance	0.079
Sandy	37.80	7.18	20.22	55.38	Count	Abundance	-
Loamy	1324.99	186.94	919.83	1803.89	µg	Fresh body mass	**0.028**
Sandy	674.33	133.36	387.49	1040.13	µg	Fresh body mass	-
Loamy	19.12	2.89	12.89	26.56	mJ h^{-1}	Community metabolism	0.051
Sandy	10.49	2.14	5.90	16.38	mJ h^{-1}	Community metabolism	-
Loamy	6.80	0.52	5.56	8.04	Count	Species richness	**0.025**
Sandy	4.57	0.54	3.26	5.88	Count	Species richness	-
Loamy	0.69	0.04	0.59	0.78	None	FEve	0.471
Sandy	0.73	0.04	0.63	0.84	None	FEve	-
Loamy	0.67	0.04	0.56	0.77	None	FDiv	0.262
Sandy	0.75	0.05	0.63	0.86	None	FDiv	-
Loamy	59.09	7.17	43.36	77.26	Proportion	Primary decomposer	0.920
Sandy	60.16	7.23	43.76	79.16	Proportion	Primary decomposer	-
Loamy	25.56	4.66	15.72	37.78	Proportion	Secondary decomposer	0.863
Sandy	24.38	4.56	14.51	36.81	Proportion	Secondary decomposer	-
Loamy	6.98	3.06	1.62	16.1	Proportion	Omnivore/predator	0.427
Sandy	2.97	2	0.09	9.86	Proportion	Omnivore/predator	-
Loamy	55.90	8.88	36.87	78.87	Proportion	Reproductive mode	**0.018**
Sandy	22.86	5.68	11.08	38.87	Proportion	Reproductive mode	-
Loamy	0.63	0.06	0.49	0.77	CWM	Pigmentation	0.516
Sandy	0.69	0.06	0.54	0.83	CWM	Pigmentation	-
Loamy	0.47	0.05	0.35	0.59	CWM	Stratification	0.140
Sandy	0.59	0.05	0.47	0.72	CWM	Stratification	-
Loamy	10.77	3.06	4.58	19.58	Proportion	Epedaphic	0.734
Sandy	9.29	2.84	3.63	17.55	Proportion	Epedaphic	-
Loamy	56.97	6.07	43.09	72.8	Proportion	Hemiedaphic	0.789
Sandy	54.60	5.94	41.03	70.11	Proportion	Hemiedaphic	-
Loamy	26.51	6.67	13.08	44.64	Proportion	Euedaphic	0.600
Sandy	21.54	6.02	9.33	38.78	Proportion	Euedaphic	-

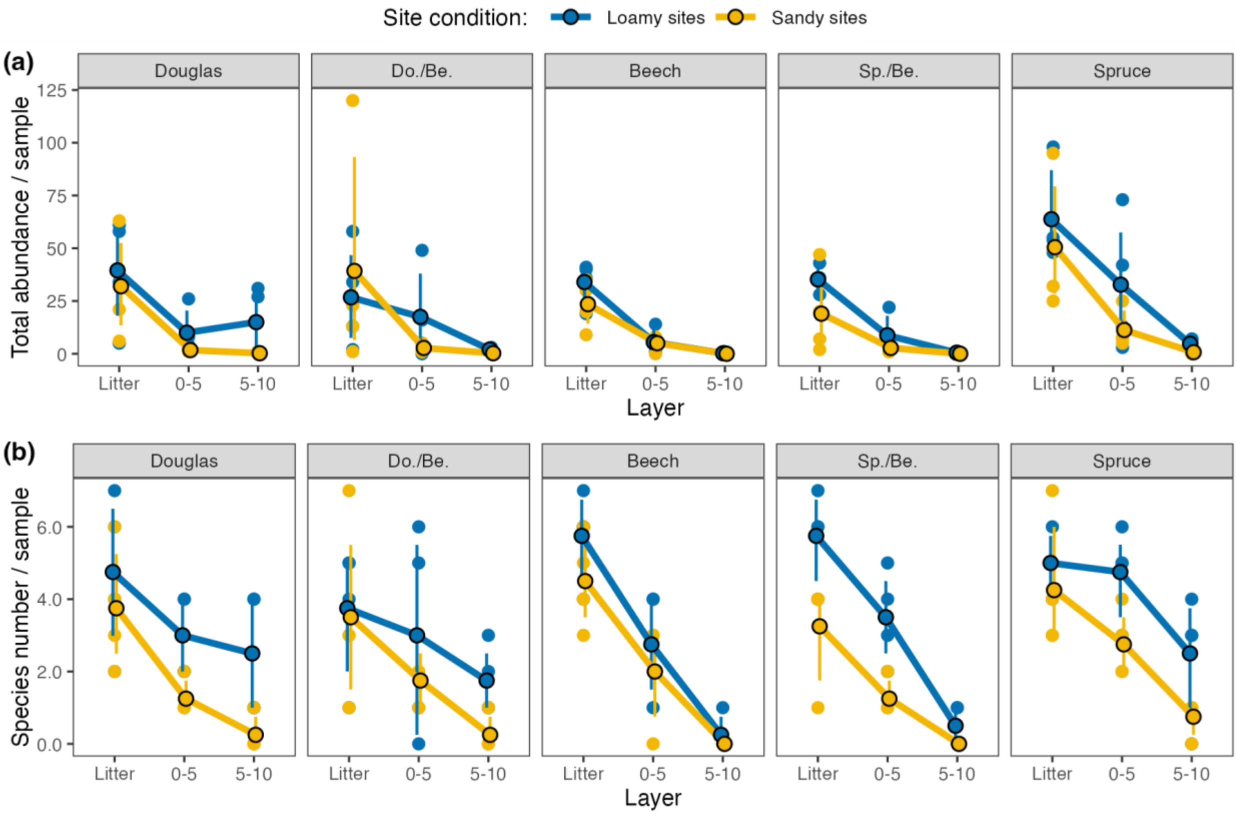

Figure S1. Abundance and species number of Collembola per sample (19.6 cm^{-2}) in the litter layer, and 0–5 and 5–10 cm soil depths at loamy and sandy sites in monocultures of Douglas fir (Douglas), European beech (Beech), Norway spruce (Spruce) and mixed stands of European beech with Douglas fir (Do./Be.) or Norway spruce (Be./Sp.). Means and 95% confidence levels.
